# Second primary malignancy among malignant solid tumor survivors aged 85 years and older

**DOI:** 10.1038/s41598-021-99260-6

**Published:** 2021-10-05

**Authors:** Zhijia Zhang, Fei Liu, Yanlin Qu, Liqian Qiu, Liqun Zhang, Qiao Yang

**Affiliations:** 1 Department of Clinical Laboratory, The Second Affiliated Hospital, Army Medical University, Chongqing, 400037 China; 2 Department of Respiratory Medicine, The 941st Hospital of the PLA Joint Logistic Support Force, Xining, 810007 China; 3 Department of Ultrasound, The 941st Hospital of the PLA Joint Logistic Support Force, Xining, 810007 China

**Keywords:** Cancer, Cancer epidemiology

## Abstract

The cancer burden in the oldest old has increased rapidly. This study aimed to investigate the epidemiology of second primary malignancy (SPM) in malignant solid tumor survivors aged 85 years and older utilizing the Surveillance, Epidemiology, and End Results (SEER) database. A total of 128,466 malignant solid tumor patients had been identified between 2000 and 2011, including 6774 patients who developed a SPM. The overall crude incidence of developing a SPM was 5.3%. Considering death as a competing event, the 3, 5, and 10-year cumulative incidence was 1.9%, 3.2%, and 5.4%, respectively. Relative younger age, male gender, surgery history, local stage and first primary malignancy (FPM) site located in the urinary system were related to higher cumulative incidence. A median time interval of 24.0 months was found between diagnosis of FPM and SPM. The most common SPM site was digestive system, whereas the least common was oral cavity and pharynx. The median overall survival (OS) was 49.0 months, and the median survival after SPM was 13.0 months. Relative older age, male gender and black race were associated with worse OS and survival after SPM, as well as higher hazard ratios of death. In conclusions, this study performed a comprehensive analysis of SPM among malignant solid tumor survivors aged 85 years and older. Additional studies are needed to characterize the specific cancer type of interest.

## Introduction

Adults aged 85 years and older, also called the oldest old, are a rapidly growing age group worldwide. This age group is expected to rise from 19 million in 2020 to 40 million by 2050 in Europe ^1^ and from 6.4 million in 2016 to 19.0 million by 2060 in the United States ^2^. The main reasons for the increase in life expectancy include less smoking, improved screening, and treatment advances ^3^.

Since cancer is a major public health problem worldwide, the burden of cancer in the oldest old has also increased accordingly ^4^. In Finland, the proportion of incident cancers in the oldest old increased to 9.6% in 2013–2017 compared to 1.5% in 1953–1957, whereas the cancer-specific mortality decreased ^5^. In the United States, there were approximately 140,690 new cancer cases and 1,944,280 cancer survivors aged 85 years and older in 2019, because the cancer-related death rate declined by 0.8% annually since 2000 ^3,6^. Moreover, an estimated 4.7 million cancer survivors among the oldest old is expected by 2040 ^6^.

Given the increased incidence and prolonged survival time, cancer survivors aged 85 years and older may suffer from a second primary malignancy (SPM). A previous study reported that approximately 16% of cancer survivors would develop second or higher-order cancers ^7^. For cancer survivors with different first primary malignancy (FPM), the probability of developing a SPM varies from 3.69 to 17.1% ^8–12^. However, few studies focus on SPM in cancer survivors aged 85 years and older.

The present study aimed to investigate the epidemiology of all SPM in patients with a malignant solid tumor as the FPM and age equals to or greater than 85 years by utilizing the Surveillance, Epidemiology, and End Results (SEER) database. Moreover, we tried to identify risk factors associated with cumulative incidence, overall survival (OS) and survival after developing a SPM.

## Results

### Baseline clinical features of patients developing a SPM

A total of 128,466 malignant solid tumor patients aged 85 years and older were identified from the SEER database between 2000 and 2011, including 6774 patients who developed a SPM. The crude incidence of developing a SPM was 5.3%. Compared to the only one primary malignancy (OPM) group, the SPM group had obviously higher proportions of patients aged 85–89 years (80% vs. 72%, *p* < 0.001), female (74% vs. 59%, *p* < 0.001), local stage (70% vs. 54%, *p* < 0.001) and surgery history (74% vs. 59%, *p* < 0.001) (Table 1). In addition, the SPM group had a higher proportion of FPM sites located in the male genital system (19% vs. 15%, *p* < 0.001), urinary system (19% vs. 12%, *p* < 0.001) and others (12% vs. 8%, *p* < 0.001). By the last follow-up date, 90% of patients with OPM and 95% of patients with SPM had died. The proportions of patients who died of cancer in the OPM and SPM cohorts were 39% and 24%, respectively. Moreover, 50% and 70% of patients died from non-cancer reasons, respectively (Table 1).

**Table 1:** Baseline clinical features comparison between OPM group and SPM group.

Variables	Total, N = 128,466 (%)	OPM, N = 121,692 (%)	SPM, N = 6774 (%)	*p value*
**Age (years)**				< 0.001
85–89	93,515 (73)	88,095 (72)	5420 (80)	
90–94	28,963 (23)	27,755 (23)	1208 (18)	
≥ 95	5988 (5)	5842 (5)	146 (2)	
**Gender**				< 0.001
Male	51,603 (40)	49,839 (41)	1764 (26)	
Female	76,863 (60)	71,853 (59)	5010 (74)	
**Race**				< 0.001
White	112,879 (88)	106,813 (88)	6066 (90)	
Black	8577 (7)	8173 (7)	404 (6)	
Others	7010 (5)	6706 (6)	304 (4)	
**FPM site**				< 0.001
Breast	23,252 (18)	22,187 (18)	1065 (16)	
Digestive system	35,093 (27)	33,517 (28)	1576 (23)	
Female genital system	6873 (5)	6610 (5)	263 (4)	
Male genital system	19,726 (15)	18,467 (15)	1259 (19)	
Oral cavity and pharynx	2915 (2)	2724 (2)	191 (3)	
Urinary system	16,328 (13)	15,041 (12)	1287 (19)	
Respiratory system	13,389 (10)	13,061 (11)	328 (5)	
Other sites	10,890 (8)	10,085 (8)	805 (12)	
**SEER stage**				< 0.001
Local	70,088 (55)	65,331 (54)	4757 (70)	
Regional	28,141 (22)	26,876 (22)	1265 (19)	
Distant	13,806 (11)	13,542 (11)	264 (4)	
Unknown	16,431 (13)	15,943 (13)	488 (7)	
**Surgery**				< 0.001
No/unknown	51,603 (40)	49,839 (41)	1764 (26)	
Yes	76,863 (60)	71,853 (59)	5010 (74)	
**Cause of death**				< 0.001
Alive	12,921 (10)	12,562 (10)	359 (5)	
Died of cancer	49,273 (38)	47,665 (39)	1608 (24)	
Died from non-cancer reasons	65,218 (51)	60,443 (50)	4775 (70)	
Died of unknown reason	1054 (1)	1022 (1)	32 (1)	

*OPM* one primary malignancy; *SPM* second primary malignancy; *FPM* first primary malignancy; *SEER* surveillance, epidemiology, and end results.

### Cumulative incidence and risk factors

**Figure 1:**
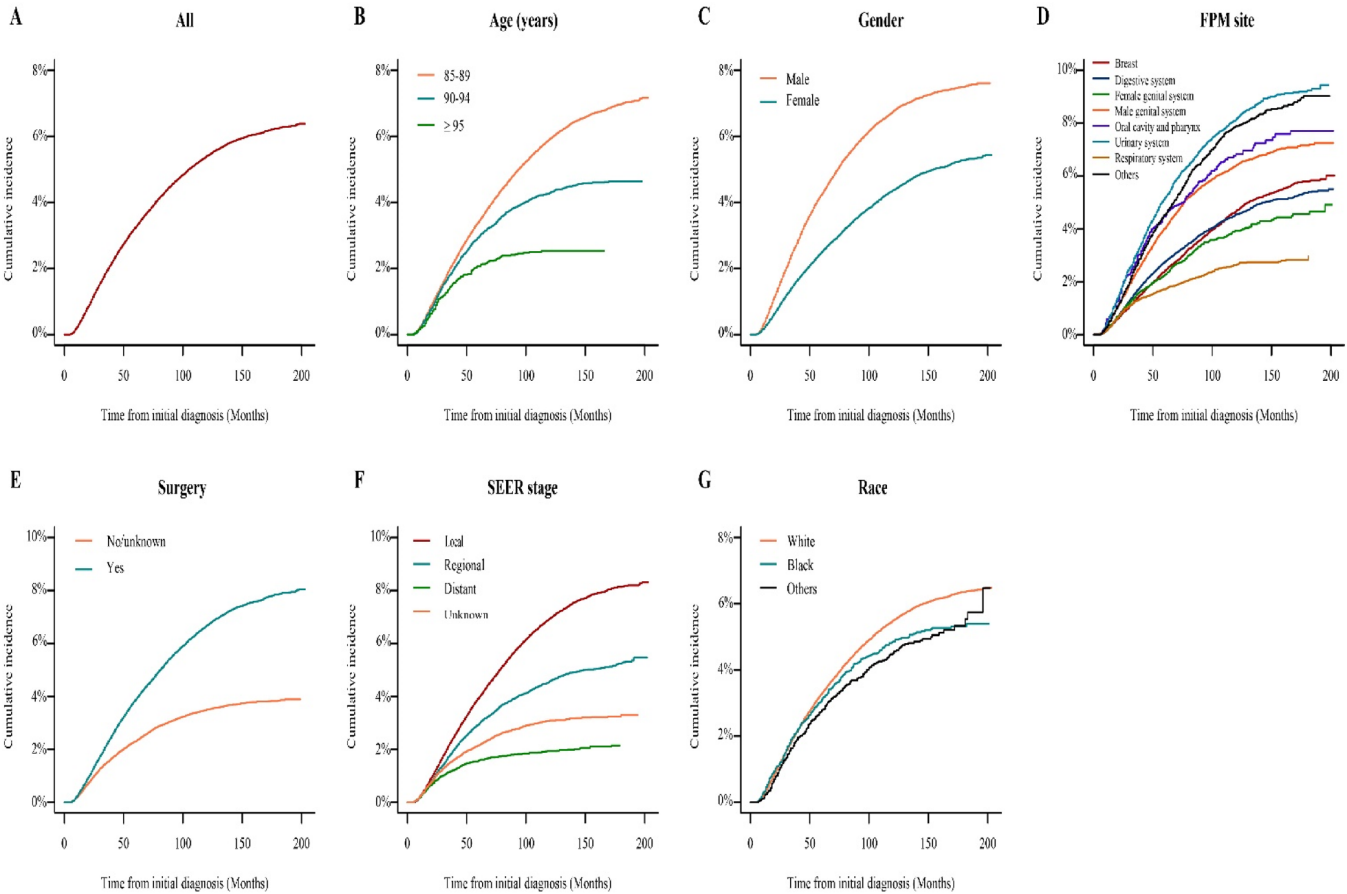
Cumulative incidence of (**A**) all and stratified by (**B**) age, (**C**) gender, (**D**) FPM site, (**E**) surgery history, (**F**) SEER stage, and (**G**) race. FPM, first primary malignancy; *SEER* surveillance, epidemiology, and end results.

Considering death as a competing event, the 3, 5, and 10-year cumulative incidence of developing a SPM was 1.9%, 3.2%, and 5.4%, respectively (Fig. 1A, Supplementary Table 1). In the subgroup analysis, patients aged 85–89 years, male, surgery history, and local stage had obviously higher cumulative incidences than counterparts (Fig. 1B, C, E, F). The cumulative incidences were similar among different races (Fig. 1G). Patients with FPM site located in the urinary system had the highest cumulative incidence, whereas those with FPM site located in the respiratory system had the lowest (Fig. 1D). The 3, 5, and 10-year cumulative incidences are listed in Supplementary Table 1. Furthermore, a forest plot was generated to display the adjusted hazards ratio (HR) and 95% confidence interval (CI) of each subgroup (Supplementary Fig. 1).

### Distribution of SPM site and time interval since index

**Figure 2:**
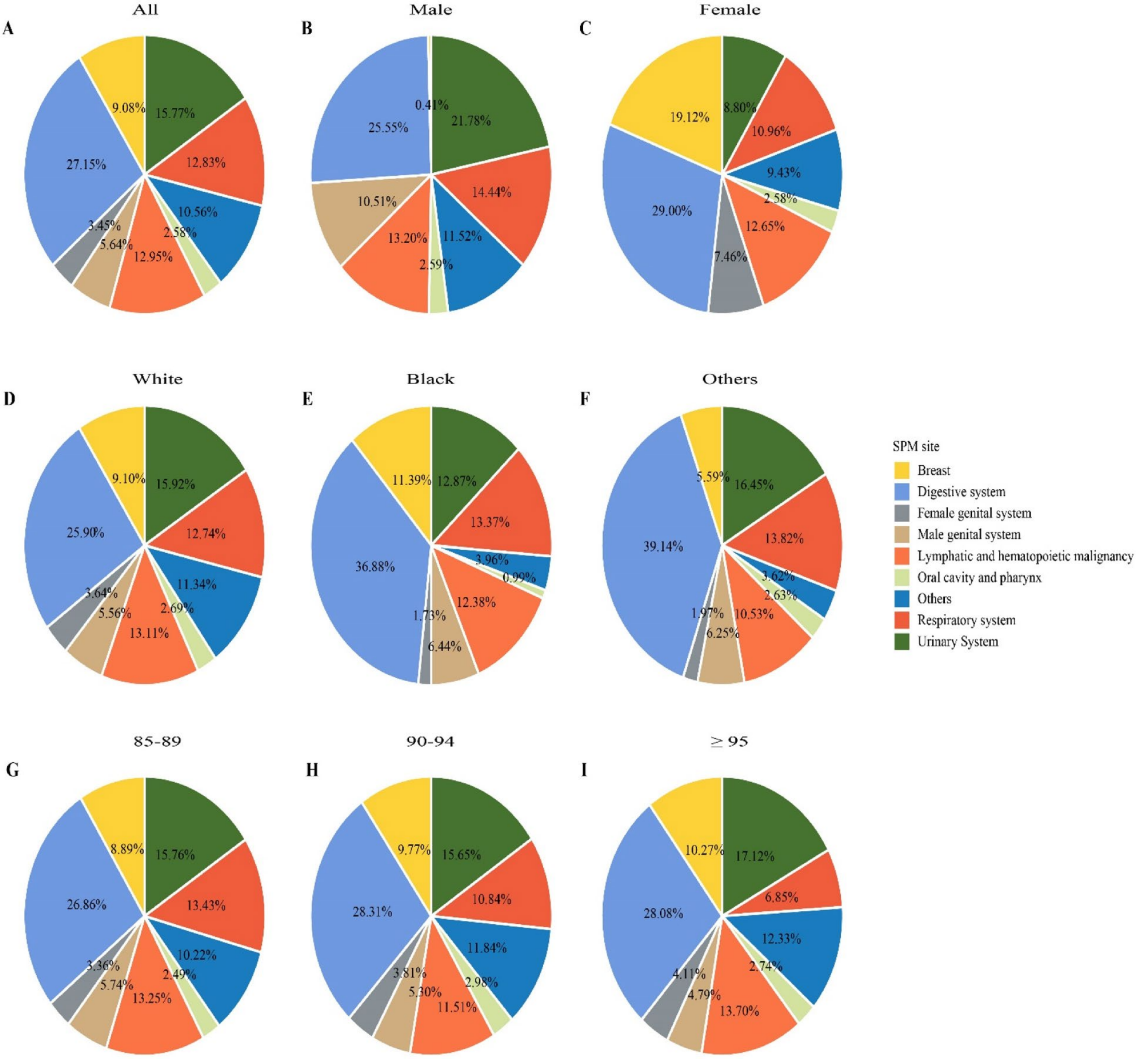
Distribution of SPM sites. (**A**) all, (**B**) male, (**C**) female, (**D**) white race, (**E**) black race, (**F**) other races, (**G**) age 85–89 years, (**H**) age 90–94 years, and (**I**) age ≥ 95 years. *SPM* second primary malignancy.

The main site of SPM was the digestive system (27.15%), followed by the urinary system (15.77%), lymphatic and hematopoietic malignancy (12.95%, including lymphoma, myeloma and leukemia), respiratory system (12.83%), other sites (10.56%), breast (9.08%), male genital system (5.64%), female genital system (3.45%) and oral cavity and pharynx (2.58%) (Fig. 2A). Compared to female patients, male patients had an obviously higher proportion of SPM sites located in the urinary system (21.78% vs. 8.8%), but much lower proportion in breast (0.41% vs. 19.12%) (Fig. 2B–C). Among different races, obvious differences in digestive system, breast and other sites were observed (Fig. 2D–F). For different age groups, the SPM site distributions were similar except that patients aged ≥ 95 years had a lower proportion of respiratory system (Fig. 2G–I).

**Figure 3:**
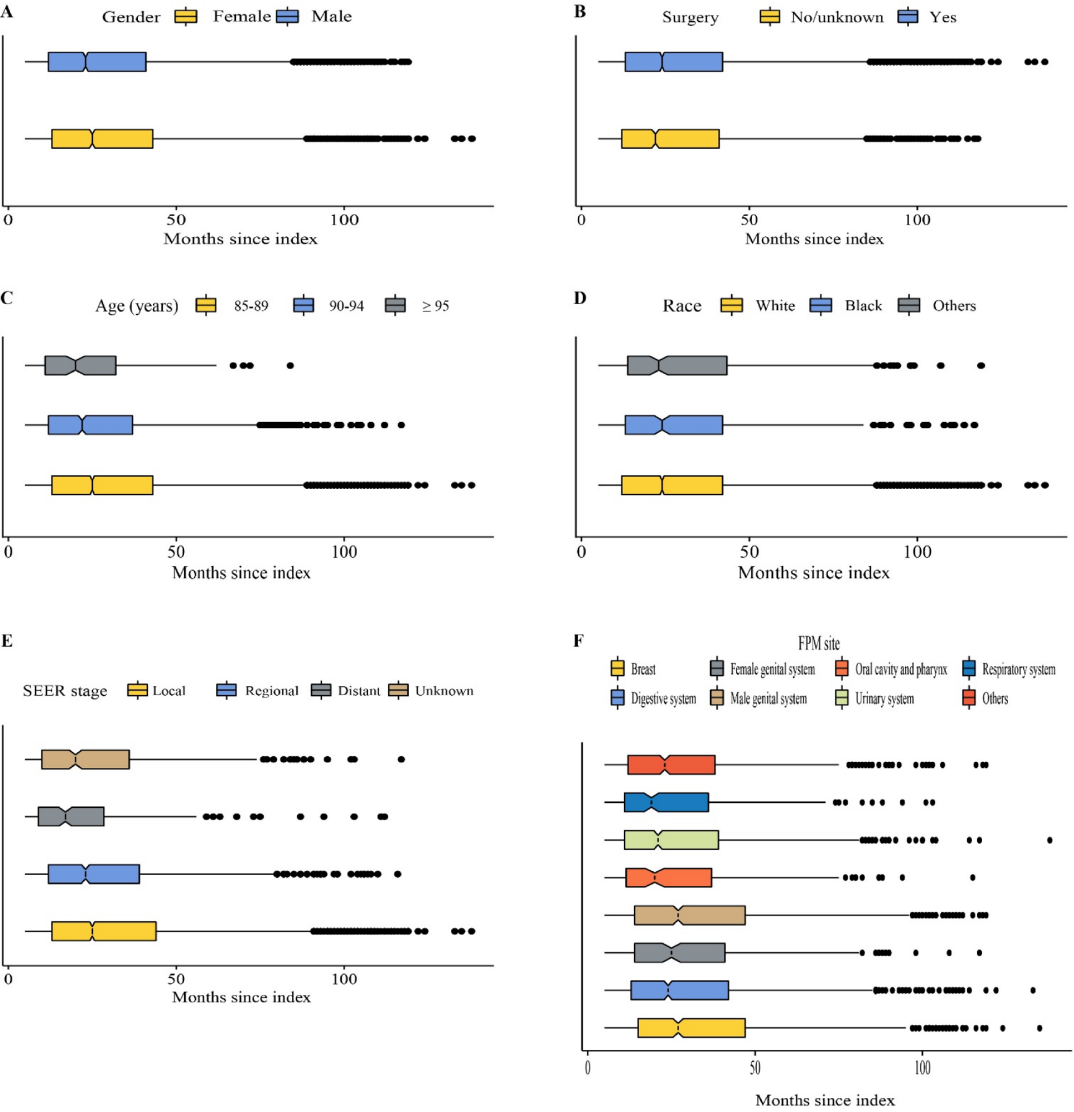
Time interval since index stratified by (**A**) gender, (**B**) surgery history, (**C**) age, (**D**) race, (**E**) SEER stage, and (**F**) FPM site. *SEER* surveillance, epidemiology, and end results; *FPM* first primary malignancy.

The median age of developing a SPM was 87 years. The median time interval since index of all patients was 24.0 months (interquartile range, 13.0–42.0 months). As shown in Fig. 3, female, surgery history, younger age and local stage were associated with longer median time interval. No obvious difference was found among different races (Fig. 3D). For different FPM sites, breast and male genital system had the longest median time interval (both 27.0 months), while respiratory system had the shortest (19.0 months) (Fig. 3F).

### Analysis of risk factors that affect overall survival

The median OS (mOS) of patients with SPM was longer than that of patients with OPM (49.0 vs. 33.0 months, HR 0.820, 95% CI 0.799–0.841, *p* < 0.001) (Supplementary Fig. 2A). To exclude the effect of baseline clinical features biases on OS, we performed a 1:5 (SPM group: OPM group) propensity score matching (PSM) analysis. No significant difference in baseline clinical features was discovered between the matched groups (Supplementary Table 2). Further survival analysis demonstrated that the mOS of patients with SPM was worse than that of patients with OPM (49.0 vs. 76.0 months, HR 1.819, 95% CI 1.769–1.871, *p* < 0.001) (Supplementary Fig. 2B).

To further explore the risk factors affecting OS in patients with SPM. Survival analysis with univariate was performed. The results turned out that all clinical features, including age, gender, race, surgery history, SEER stage, FPM site and SPM site, were related to OS. The survival plot of each variable is shown in Supplementary Fig. 3. Furthermore, we conducted a multivariate Cox analysis to evaluate the adjusted risk factors that affected OS. The results showed that patients with older age, male gender, black race, advanced stage and no/unknown surgery history had higher HRs for death (Supplementary Fig. 4). Compared to FPM site of breast, those of oral cavity and pharynx (HR 1.198, 95% CI 1.013–1.416, *p* = 0.035), respiratory system (HR 1.154, 95% CI 1.004–1.328, *p* = 0.044) and urinary system (HR 1.178, 95% CI 1.064–1.305, *p* = 0.002) had higher risks of death. Other FPM sites had no significant difference. Compared to SPM site of breast, all other SPM sites had higher risks of death, of which SPM site of respiratory system had the highest risk (HR 2.129, 95% CI 1.900–2.386, *p* < 0.001) (Supplementary Fig. 4).

### Survival after developing a SPM

**Figure 4:**
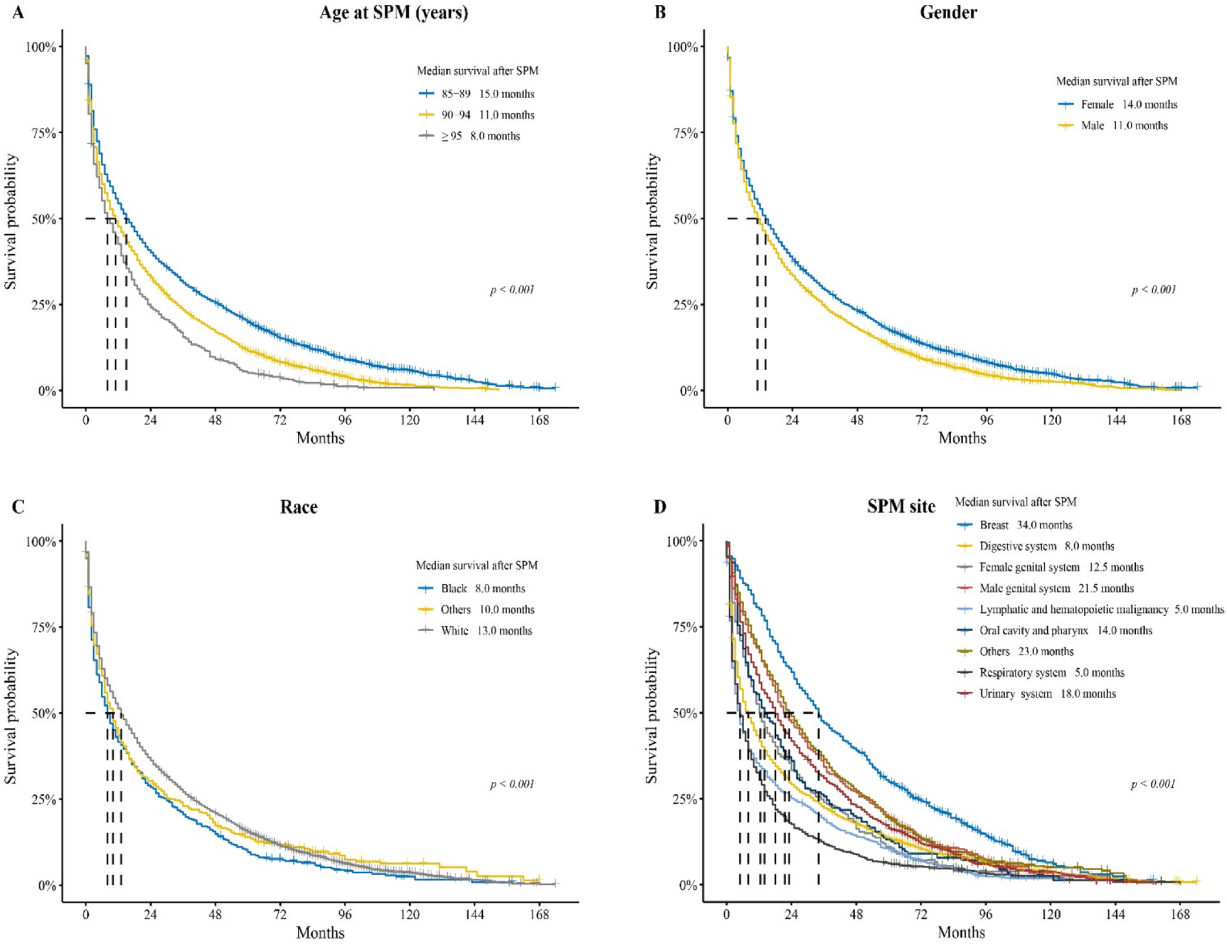
Analysis of survival after developing a SPM stratified by (**A**) age at SPM, (**B**) gender, (**C**) race, and (**D**) SPM site. SPM, second primary malignancy.

**Figure 5:**
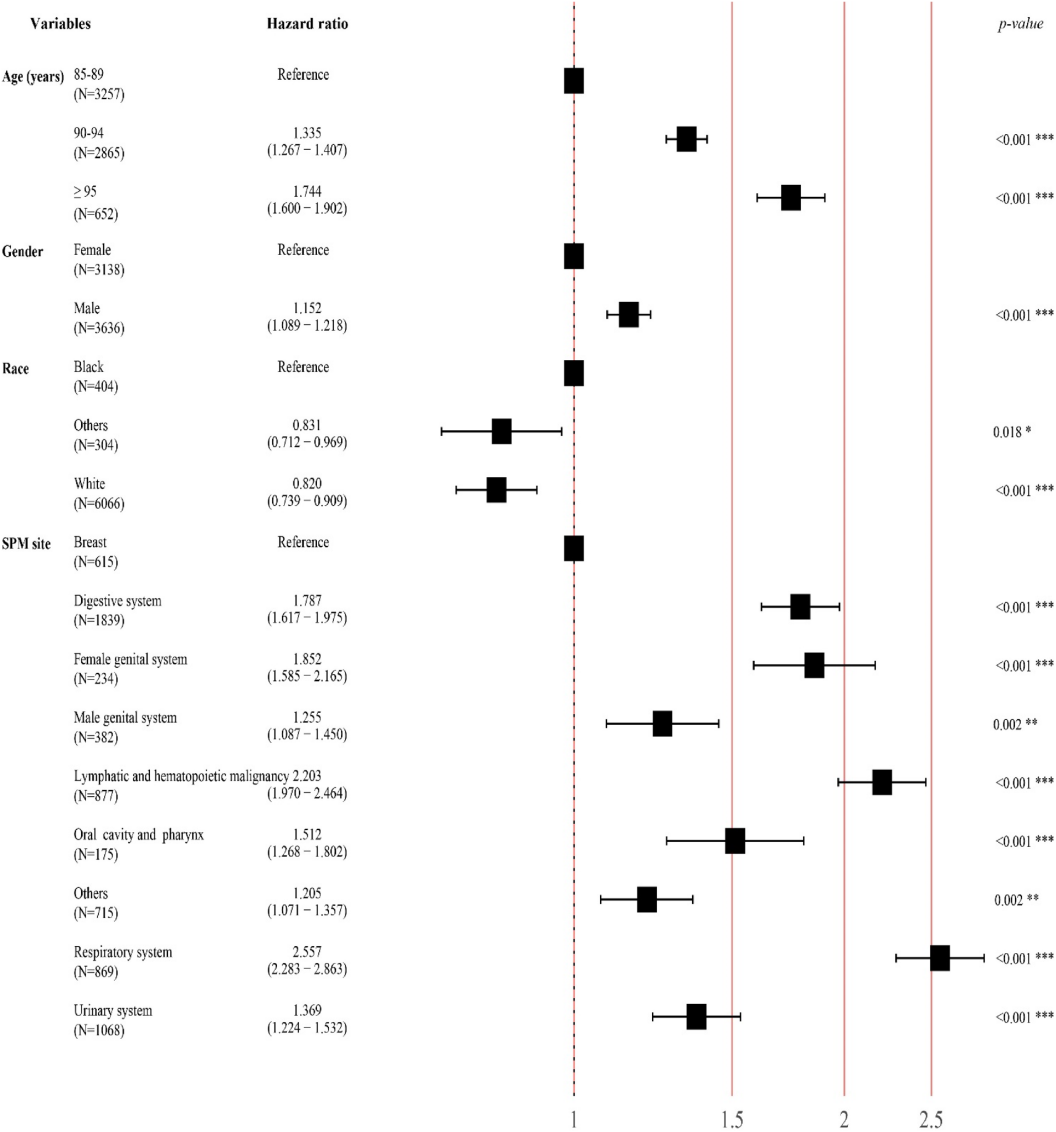
A forest plot displaying the HR and 95% CI of each variable affecting survival after SPM. The square and line segments represent the HRs and 95% CI, and HR > 1.000 indicates a higher risk. HR, hazards ratio; CI, confidence interval; SPM, second primary malignancy.

We further investigated the survival after developing a SPM. The median survival after SPM of the entire population was 13.0 months. For patients developing a SPM at age 85–89 years, the median survival was 15.0 months, which was better than that of patients developing a SPM at age 90–94 years (11.0 months) and ≥ 95 years (8.0 months) (*p* < 0.001, Fig. 4A). The HR was 1.335 (95% CI 1.267–1.407, *p* < 0.001) and 1.744 (95% CI 1.600–1.902, *p* < 0.001) (Fig. 5). Female patients had a better median survival after SPM compared to male patients (14.0 vs. 11.0 months, *p* < 0.001; HR 1.152, 95% CI 1.089–1.218, *p* < 0.001) (Figs. 4B and 5). Patients of black race had a median survival of 8.0 months after SPM, which was worse than that of white race (13.0 months, HR 0.831) and other races (10.0 months, HR 0.820) (*p* < 0.001, Figs. 4C and 5). For different SPM sites (Fig. 4D), patients with a SPM located in breast had the best median survival after SPM (34.0 months). While those with a SPM located in the respiratory system or lymphatic and hematopoietic malignancy had the worst median survival after SPM (both 5.0 months). Compared to breast, all other sites had higher HRs for death. Respiratory system had the highest HR for death (2.557, 95% CI 2.283–2.863, *p* < 0.001) (Fig. 5).

## Discussion

In this large population-based study, we performed a comprehensive analysis of malignant solid tumor survivors aged 85 years and older with a SPM. The overall crude incidence of developing a SPM in this population was 5.3%, 6774 out of 128,466 patients developed a SPM between 2000 and 2011. A previous study ^13^ reported that approximately 6.9% in 10 most common cancer survivors aged 80 years and older developed a SPM, which was slightly higher than that of our report. Compared to young patients, old patients had a lower risk of developing a SPM ^13,14^. The 10-year cumulative incidence of developing a SPM was 5.4% in this study, which was similar to that among adolescent and young adult (AYA) survivors of cancer ^14^.

Considering death as a competing event, relative younger age (85–89 years) at FPM diagnosis, male patients, receiving surgery treatment for FPM, local stage and FPM site located in the urinary system were associated with higher cumulative incidence in the oldest old patients. In addition to surgery, radiotherapy and chemotherapy were also proved to be associated with an increased risk of developing a SPM ^15–17^. Moreover, a review demonstrated that a family history of cancer, genetic variants, tobacco, alcohol, obesity, etc. were also etiological factors of SPM in cancer survivors ^18^.

Donin and colleagues reported that the most common SPM was lung cancer (18%), followed by colorectal cancer (12%), prostate cancer (9%), and bladder cancer (8%) in cancer survivors aged ≥ 18 years ^13^. Two other studies also found lung cancer as the most common SPM in adults ^19,20^. Among the survivors of AYA cancer, the most common SPM was breast cancer (32%), followed by melanoma (14%) and ovarian cancer (5%) ^14^. A population-based study from Switzerland reported that the most common SPM was prostate cancer (28.5%) in males and breast cancer in females ^21^. In this study, we analyzed the distribution of SPM stratified by system location instead of specific cancer. Among malignant solid tumor survivors in the oldest old, SPM site located in digestive system (27.15%), urinary system (15.77%), lymphatic and hematopoietic malignancy (12.95%) and respiratory system (12.83%) were the most common.

Several interesting findings were demonstrated in the current study. First, though female patients had better OS than male patients, the cumulative incidence was lower in female patients compared to male patients (HR 0.713, 95% CI 0.670–0.758). The probable reason was the longer time interval since index in female patients. The survival after SPM was also better in female patients. A potential explanation could be that a higher proportion of SPM was located in the breast, which was demonstrated to have the best survival after SPM. Whereas higher tumor burden (shorter time interval to develop a SPM) and higher proportions of a SPM located in the respiratory system (only a median survival of 5.0 months after SPM) and digestive system (5.0 months) led to worse OS and survival after SPM in male patients.

Second, though patients aged 95 years and older had shorter time interval since index compared to the other two age groups, the cumulative incidence remained lower in this age group. Complicated comorbidities, functional disabilities and poor nutritional status could put patients aged 95 years and older at a high risk of non-cancer-related death, or contraindicated to receive cancer treatment such as chemotherapy and radiation ^5^. These could result in short life expectancy and insufficient time to develop a SPM. These factors may also explain why the survival after SPM was worse in patients aged 95 years and older in the case of similar SPM site distribution.

Third, the OS was better in the SPM cohort than in the OPM cohort before PSM. The probable reasons might be the higher proportions of relative younger age and local stage at first cancer diagnosis were observed in the SPM cohort, which were related to survival benefit. While, after balancing the baseline clinical features, the OS was better in the OPM cohort. Especially after approximately 24.0 months, which was similar with the median time interval since index of developing a SPM, the survival curves between these two groups separated significantly.

Both the FPM site and SPM site had certain impact on survival. Patients with breast as FPM site or SPM site had the best OS or survival after SPM, whereas patients with respiratory system as FPM site or SPM site had the worst. Moreover, relative older age, male gender and black race were associated with worse OS and survival after SPM, as well as higher HRs of death. Currently, no standard guidelines are available for the treatment of patients with SPM ^15^. In general, the treatment strategy should consider both FPM and SPM, stage of the disease and health status. Individual treatment followed by a multidisciplinary team assessment should be considered. Given that tobacco, alcohol and obesity contribute to the incidence of SPM, drinking and smoking cessation, and keeping fit could prevent the incidence to some extent ^18^. Moreover, some of the oldest old have contraindications to aggressive curative therapy, so palliative care should be considered in such patients ^22,23^.

This study had some limitations. First, radiotherapy and chemotherapy information were not included in this study, because information about these two variables are incomplete in the SEER database, which might lead to some deviation. Second, the SEER database lacks information on smoking history, alcohol history and body mass index, which could help to better describe the profile of SPM in the oldest old patients. Next, we did not construct a visual nomogram to predict the probability of developing a SPM, primarily because the overall incidence in this population was very low. Finally, we did not characterize the specific cancer type in this study. Further studies should be performed to offer insights on the specific cancer type of interest.

## Conclusion

To the best of our knowledge, this is the first population-based study focusing on SPM of malignant solid tumor survivors aged ≥ 85 years. The results turned out approximately 5.3% patients would develop a SPM in the population. Relative younger age, male gender, surgery history, local stage and first primary malignancy (FPM) site located in the urinary system were related to higher cumulative incidence. The development of a SPM would rapidly decrease the life expectancy, with a median survival of 13.0 months after SPM. Therefore, it is important to identify high-risk groups and rationally adjust the treatment strategy. This study offered a comprehensive profile about SPM among malignant solid tumor survivors aged 85 years and older, which could provide an evidence for prevention, screening and survival recommendations for this specific ages. Additional studies are needed to characterize the specific cancer type of interest.

## Methods

### Study population and variables

We extracted data from SEER 18 registries, which was released on September 2, 2020. We identified all malignant solid tumor patients aged ≥ 85 years. Only patients with FPM diagnosis between 2000 and 2011 were included to ensure 5 years of follow-up. The variables include age (85–89, 90–94, ≥ 95 years), gender (male, female), race (white, black, others), SEER stage (local, regional, distant, unknown), surgery (yes, no/unknown) and FPM site (breast, digestive system, female genital system, male genital system, oral cavity and pharynx, urinary system, respiratory system, other sites). For variable surgery, “yes” means a surgery treatment to the primary tumor site, while the specific surgery method is not detailed. Besides, 1592 out of 128,466 (1.2%) patients had unknown surgery history. Hence, patients with no or unknown surgery history were integrated one subgroup, i.e. no/unknown.

### Outcome measurement

FPM was defined as the firstly confirmed primary malignancy. SPM was defined as the secondly confirmed primary malignancy in patients with FPM. The SEER database uses a set of multiple primaries rules to distinguish SPM from recurrence, in case of the same organ records in both FPM and SPM ^24^. Time interval since index was defined as time interval between diagnosis of FPM and diagnosis of SPM. The time interval between SPM and FPM was at least 6 months. OPM was defined as only one primary malignancy was confirmed in patients until the last follow-up. OS was defined as the follow-up time from diagnosis of FPM to death due to any reason. Survival after SPM was defined as the follow-up time from diagnosis of SPM to death due to any reason. Patients who were alive at the last follow-up on 31 December 2016 were regarded as censored cases.

### Study design and statistical analysis

Pearson’s chi-square test was used to compare the differences in clinical features between patients with OPM and patients with SPM. Regarding patient death as a competing event, Fine and Gray model was used to calculate the cumulative incidence of developing a SPM and estimate the HR and 95% CI conditioned on the variables of interest, including age, gender, race, FPM site, SEER stage and surgery history. Then, the differences in the distribution of SPM site and time interval since index among subgroups were analyzed.

Survival analyses were performed by the Kaplan–Meier method and the log-rank test. Multivariate Cox proportional hazards model was used to calculate adjusted HR and 95% CI that affected OS and survival after SPM. PSM was used to match each SPM patient with five OPM patients for further survival analysis. The following predetermined variables were considered for matching, including age, gender, race, surgery history, SEER stage and FPM site.

All cases were exported from SEER*Stat software (version 8.3.8; https://seer.cancer.gov/seerstat/). Two-sided *p*-value < 0.05 was considered to indicate statistically significant difference. All statistical analyses and PSM (MatchIt package) were performed with R software (version 4.0.3; http://www.r-project.org/).

## Supplementary Information

Supplementary Information 1.

Supplementary Information 2.

Supplementary Information 3.

Supplementary Information 4.

Supplementary Information 5.

Supplementary Information 6.

## Data Availability

The datasets for this study can be obtained from the corresponding author upon any reasonable request.
