# Supplementary material for: Second primary malignancy among malignant solid tumor survivors aged 85 years and older
Source: Sci Rep. 2021 Oct 5;11:19748. doi: 10.1038/s41598-021-99260-6 (PMC8492691; doi:10.1038/s41598-021-99260-6)
Supplement: Supplementary file 5 — Supplementary Information 5. [file 41598_2021_99260_MOESM5_ESM.docx]

**Supplementary Figure legends**

Supplementary Figure 1. A forest plot displaying the HR and 95% CI of each variable affecting cumulative incidence of developing a SPM based on Fine and Gray model.

HR, hazards ratio; CI, confidence interval; SPM, second primary malignancy; FPM, first primary malignancy; SEER, Surveillance, Epidemiology, and End Results.

Supplementary Figure 2. OS analysis between OPM and SPM before A) and after B) propensity score matching.

OS, overall survival; OPM, one primary malignancy; SPM, second primary malignancy; HR, hazards ratio; CI, confidence interval.

Supplementary Figure 3. OS analysis stratified by A) gender, B) age, C) race, D) surgery history, E) SEER stage, F) FPM site and G) SPM site.

OS, overall survival; SEER, Surveillance, Epidemiology, and End Results; FPM, first primary malignancy; SPM, second primary malignancy.

Supplementary Figure 4. A forest plot displaying the HR and 95% CI of each variable affecting OS. The square and line segments represent the HRs and 95% CI, and HR > 1.000 indicates a higher risk.

HR, hazards ratio; CI, confidence interval; OS, overall survival; SEER, Surveillance, Epidemiology, and End Results; FPM, first primary malignancy; SPM, second primary malignancy.
