# Supplementary material for: Second primary malignancy among malignant solid tumor survivors aged 85 years and older
Source: Sci Rep. 2021 Oct 5;11:19748. doi: 10.1038/s41598-021-99260-6 (PMC8492691; doi:10.1038/s41598-021-99260-6)
Supplement: Supplementary file 6 — Supplementary Information 6. [file 41598_2021_99260_MOESM6_ESM.docx]

Supplementary Table 1: Cumulative incidence.

| **Variables** | **3-year (%)** | **5-year (%)** | **10-year (%)** |
| --- | --- | --- | --- |
| **All** | 1.9 | 3.2 | 5.4 |
| **Age (years)** |  |  |  |
| 85-89 | 2.0 | 3.4 | 5.9 |
| 90-94 | 1.8 | 2.9 | 4.3 |
| ≥ 95 | 1.4 | 2.1 | 2.5 |
| **Gender** |  |  |  |
| Male | 2.5 | 4.2 | 6.8 |
| Female | 1.5 | 2.5 | 4.4 |
| **Race** |  |  |  |
| White | 2.0 | 3.3 | 5.5 |
| Black | 2.0 | 3.1 | 4.9 |
| Others | 1.7 | 2.8 | 4.5 |
| **FPM site** |  |  |  |
| Breast | 1.3 | 2.4 | 4.7 |
| Digestive system | 1.6 | 2.8 | 4.5 |
| Female genital system | 1.5 | 2.3 | 3.9 |
| Male genital system | 2.4 | 4.0 | 6.4 |
| Oral cavity and pharynx | 2.7 | 4.5 | 6.8 |
| Urinary system | 3.0 | 5.2 | 8.1 |
| Respiratory system | 1.3 | 1.7 | 2.6 |
| Others | 2.6 | 4.5 | 7.9 |
| **SEER stage** |  |  |  |
| Local | 2.3 | 3.9 | 6.9 |
| Regional | 1.8 | 3.0 | 4.6 |
| Distant | 1.2 | 1.6 | 1.9 |
| Unknown | 1.5 | 2.2 | 3.1 |
| **Surgery** |  |  |  |
| No/unknown | 1.5 | 2.3 | 3.5 |
| Yes | 2.2 | 3.9 | 6.7 |
| FPM, first primary malignancy; SEER, Surveillance, Epidemiology, and End Results. | | | |

Supplementary Table 2: Clinical features comparison between SPM group and matched OPM group.

| **Variables** | **Total,**  **N=40644 (%)** | **Matched OPM,**  **N=33870 (%)** | **SPM,**  **N=6774 (%)** | ***p-value*** |
| --- | --- | --- | --- | --- |
| **Age (years)** |  |  |  | *0.947* |
| 85-89 | 32557 (80) | 27137 (80) | 5420 (80) |  |
| 90-94 | 7231 (18) | 6023 (18) | 1208 (18) |  |
| ≥ 95 | 856 (2) | 710 (2) | 146 (2) |  |
| **Gender** |  |  |  | *0.940* |
| Male | 21796 (54) | 18160 (54) | 3636 (54) |  |
| Female | 18848 (46) | 15710 (46) | 3138 (46) |  |
| **Race** |  |  |  | *0.931* |
| White | 36433 (90) | 30367 (90) | 6066 (90) |  |
| Black | 2384 (6) | 1980 (6) | 404 (6) |  |
| Others | 1827 (4) | 1523 (4) | 304 (4) |  |
| **FPM site** |  |  |  | *1.000* |
| Breast | 6398 (16) | 5333 (16) | 1065 (16) |  |
| Digestive system | 9453 (23) | 7877 (23) | 1576 (23) |  |
| Female genital system | 1587 (4) | 1324 (4) | 263 (4) |  |
| Male genital system | 7552 (19) | 6293 (19) | 1259 (19) |  |
| Oral cavity and pharynx | 1129 (3) | 938 (3) | 191 (3) |  |
| Urinary system | 7713 (19) | 6426 (19) | 1287 (19) |  |
| Respiratory system | 1996 (5) | 1668 (5) | 328 (5) |  |
| Other sites | 4816 (12) | 4011 (12) | 805 (12) |  |
| **SEER stage** |  |  |  | *0.992* |
| Local | 28539 (70) | 23782 (70) | 4757 (70) |  |
| Regional | 7605 (19) | 6340 (19) | 1265 (19) |  |
| Distant | 1601 (4) | 1337 (4) | 264 (4) |  |
| Unknown | 2899 (7) | 2411 (7) | 488 (7) |  |
| **Surgery** |  |  |  | *0.876* |
| No/unknown | 10618 (26) | 8854 (26) | 1764 (26) |  |
| Yes | 30026 (74) | 25016 (74) | 5010 (74) |  |
| OPM, one primary malignancy; SPM, second primary malignancy; FPM, first primary malignancy; SEER, Surveillance, Epidemiology, and End Results. | | | | |
